# Dynamic Modulation of THz Absorption Frequency, Bandwidth, and Amplitude via Strontium Titanate and Graphene

**DOI:** 10.3390/nano12081353

**Published:** 2022-04-14

**Authors:** Tong Wu, Guan Wang, Yang Jia, Yabin Shao, Yang Gao, Yachen Gao

**Affiliations:** 1 Department of Optoelectronic Information, Electronic Engineering College, Heilongjiang University, Harbin 150080, China; 124wutong@163.com (T.W.); wang2687220886@163.com (G.W.); yang_1990@163.com (Y.J.); 2 School of Jia Yang, Zhejiang Shuren University, Shaoxing 312028, China; shao_yabin@163.com

**Keywords:** terahertz, multi-functional, absorber, broadband absorption

## Abstract

A multi-functional broadband absorber based on graphene and strontium titanate (STO) film was designed. Additionally, the frequency, bandwidth, and amplitude of the absorber could be tuned by adjusting temperature and Fermi level of the graphene. By using the finite-difference time-domain (FDTD) method, the numerical calculation result shows that, when keeping the device temperature at 230 K and setting graphene Fermi level to be 1 eV, three absorption peaks at 1.72 THz, 2.08 THz, and 2.59 THz were realized and combined into a broadband absorption from 1.68 to 2.74 THz. As the STO temperature was increased from 230 K to 310 K, the center frequency moved from 2.2 THz to 2.45 THz; correspondingly, the broadband absorption range was widened from 1.06 THz to 1.24 THz. When the temperature was fixed at 230 K and the graphene Fermi level was tuned from 1 eV to 0.7 eV, the absorption bandwidth decreased from 1.06 THz to 0.64 THz. While the Fermi level was tuned continually to be 0.01 eV, only a single absorption peak with an absorption rate of 0.29 existed. The broadband absorption and tuning mechanism of the absorber were analyzed using impedance matching theory. Furthermore, we also studied the effect of incident angle and polarization direction on the properties of the absorber. The multi-functional tunable absorber provides potential applications for the design of more efficient terahertz functional devices in the future.

## 1. Introduction

Recently, terahertz devices have received widespread attention due to their various applications in communication, imaging, sensing, detecting, and so on [1,2,3,4,5]. Among them, the absorber, as an important part of the optical control device, still needs more in-depth research. However, because of the limitations of natural materials in the terahertz band, it is necessary to find new strategies to design efficient absorbers. With the advancement of micro/nanofabrication techniques, metamaterials have attracted researchers’ attention [6]. Especially, absorbers based on metamaterials have been developed rapidly. Up to now, in order to meet different practical application requirements, researchers have realized narrow-band, multi-band, and broadband absorption in the terahertz band by designing structures such as split rings, I-type, notched rings, and crosses structures [7,8,9,10,11,12,13]. However, because of the fixed absorption performance of many proposed absorbers, the application fields of them are limited.

The development of tunable absorbers breaks this limitation, which makes various parameters of the absorber be tuned via electronic control [14,15,16,17,18,19], optical control [20,21,22,23], and temperature control [24,25,26,27]. Moreover, many researchers hope to achieve dual control effects in a single device. To design absorbers using a combination of two kinds of tunable materials is one of the strategies. For example, in 2019, Xin Huang et al. proposed an absorber which can realize the tuning of absorption amplitude by changing the Fermi level of the graphene sheet and the shifting of peak frequency by changing the STO temperature [28]. In 2020, Tongling Wang et al. designed an absorber consisting of graphene and vanadium dioxide (VO_2_). When changing the Fermi level of graphene independently, the absorption can be tuned and when changing the temperature of device, the conversion between absorption and reflection characteristics of the device is achieved [29]. In 2020, Han Xiong et al. used STO and bulk Dirac semimetal (BDS) to design bifunctional absorbers. They realized the modulation of peak frequency and intensity by changing the STO temperature and BDS Fermi energy [30,31]. In the same year, Zhaoxin Li realized the tuning of the frequency and intensity of the three absorption peaks by adjusting the Fermi level of the Dirac semimetal and VO_2_ temperature in the absorber, respectively [32]. In 2021, Liu et al. proposed a switchable bifunctional metamaterial based on STO and graphene. By changing the Fermi level of graphene, the absorption bandwidth can be changed from broadband to narrowband. When changing the STO temperature, the frequency of the absorption peak can be tuned [33]. However, the design of an efficient triple-function tunable absorber remains a challenge.

In this paper, a structure consisting of patterned graphene and STO film is proposed to realize the modulation of center frequency, bandwidth, and intensity of the absorber. Via the FDTD method, the properties of the absorber were studied. Based on electric field, charge distribution, and impedance matching theory, the absorption and tunable mechanism of the absorber were analyzed theoretically. In addition, the relationship between absorption performance and oblique incident angles was also investigated.

## 2. Structure Design and Method

**Figure 1:**
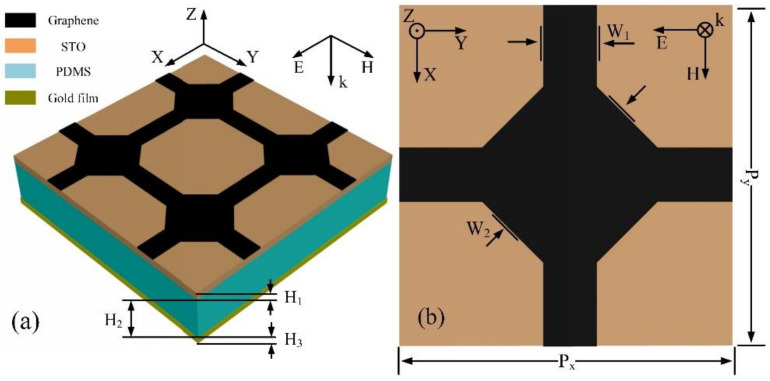
(**a**) Schematic of tunable absorber, consisting of patterned graphene, STO, PDMS, and gold film. (**b**) Top view of tunable absorber.

The proposed absorber is shown in Figure 1, where Figure 1a is three-dimensional structure and Figure 1b is the top view of the unit cell. The polydimethylsiloxane (PDMS) with thickness of $H_{2}=12.2\;\mu m$ was embedded between the STO film and gold film. The thickness of gold film was $H_{3}=0.2\;\mu m$. As shown in Figure 1b, each pattern graphene on the top of STO film was a combination of octagonal structure graphene and graphene strips with dimensions of $W_{1}=1.5\;\mu m$, $W_{2}=5.6\;\mu m$. As a loss metal, the gold film had conductivity of $4.56\times 10^{7}\;{\mathrm{Sm}}^{-1}$ [34]. The relative permittivity of PDMS was set to be 2.35+0.141 i [35].

According to random-phase approximation (RPA) theory, the conductivity $\sigma_{g}$ of graphene can be expressed as the combining of intraband $\sigma_{\mathrm{int}ra}$ conductivity and interband $\sigma_{\mathrm{int}er}$ conductivity [36]:

(1) $$\begin{array}{c} \sigma_{g}=\sigma_{\mathrm{int}ra}+\sigma_{\mathrm{int}er}=\frac{2e^{2}k_{B}T}{\pi \hbar^{2}}\frac{i}{\omega +i\tau^{-1}}ln\left(2cosh\left(\frac{E_{F}}{2k_{B}T}\right)\right)+\\ \frac{e^{2}}{4\pi \hbar }\left[\frac{1}{2}+\frac{1}{\pi }arctan\left(\frac{\hbar \omega -2E_{F}}{2k_{B}T}\right)-\frac{i}{2\pi }ln\frac{{\left(\hbar \omega +2E_{F}\right)}^{2}}{{\left(\hbar \omega +2E_{F}\right)}^{2}+4{\left(k_{B}T\right)}^{}}\right] \end{array}$$

where e is the electron charge, $k_{B}$ is the Boltzmann constant, ℏ is the reduced Planck constant, T is the operation temperature, ω is the angular frequency of the terahertz wave,τ is the carrier relaxation time, and $E_{F}$ is the Fermi level.

STO has the characteristics of high permittivity, low dielectric loss, and good chemical stability. Furthermore, in the terahertz band, the permittivity of STO is sensitive to temperature, so it is very suitable for designing terahertz tunable devices [37]. The temperature-dependent relative permittivity in the THz band is expressed as follows [38]:

(2) $$\varepsilon_{\omega }=\varepsilon_{\infty }+\frac{f}{\omega_{0}-\omega^{2}-i\omega \gamma }$$

where $\varepsilon_{\infty }=9.6$ represents the high-frequency bulk permittivity, $f=2.36\times 10^{6}{\;\mathrm{cm}}^{2}$ is an oscillator strength depending on temperature, and γ is the damping factor. In addition, the $\omega_{0}$ and γ can be expressed as:

(3) $$\begin{array}{l} \omega_{0}(T)\left[{\mathrm{cm}}^{-1}\right]=\sqrt{31.2(T-42.5)}\\ \gamma (T)\left[{\mathrm{cm}}^{-1}\right]=-3.3+0.094T \end{array}$$

where T is temperature of STO, and $\omega_{0}$ and ω are the soft mode frequency and angular frequency, respectively. Therefore, the relative permittivity of STO can be calculated by angular frequency f and temperature T.

**Figure 2:**
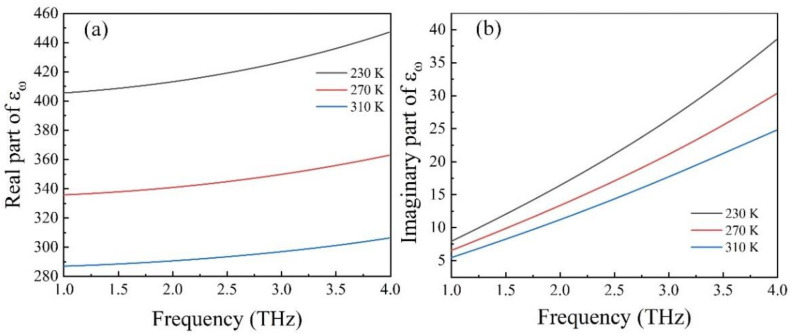
The (**a**) real and (**b**) imaginary parts of the STO permittivity.

Figure 2a,b shows the real and imaginary parts of the permittivity of STO at different temperatures. We can see that, at the certain temperature, when frequency increased from 1 THz to 4 THz, the real and imaginary parts of the permittivity increased. When the frequency was fixed, the real and imaginary parts of permittivity decreased with the increase of temperature, and the decreasing trend of the real part was more obvious than that of the imaginary part.

In this work, the electromagnetic properties of absorber could be studied based on FDTD algorithm (software: FDTD solution). The absorption rate was calculated via formula: $A\left(\omega \right)=1-\left|R\left(\omega \right)\right|-\left|T\left(\omega \right)\right|=1-\left|S_{11}{\left(\omega \right)}^{2}\right|-\left|S_{21}{\left(\omega \right)}^{2}\right|$. $T\left(\omega \right)$ and $R\left(\omega \right)$ are the transmittance and reflectance obtained by S-parameter $S_{21}(\omega )$ and $S_{11}(\omega )$, respectively [39]. Because the gold film under PDMS was thicker than the skin depth of terahertz wave, the transmission of the absorber was set to be $T\left(\omega \right)=S_{21}{\left(\omega \right)}^{2}=0$.

## 3. Results and Discussion

**Figure 3:**
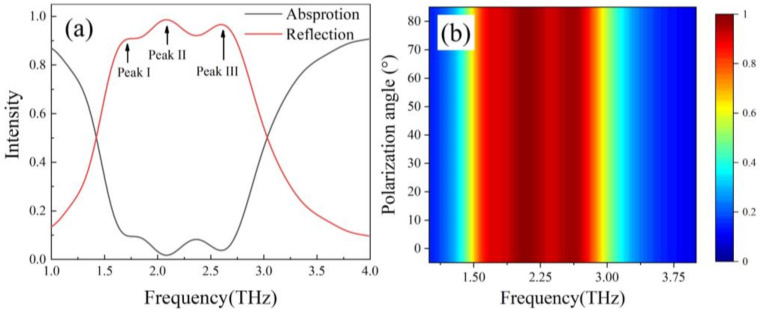
(**a**) The absorption and reflection rate of absorber. (**b**) The absorption rate under different polarization of the incident wave.

Firstly, the temperature of absorber and the Fermi level of patterned graphene were set to be 230 K and 1 eV, respectively. Under normal incident plane wave, the reflection (black line) and absorption (red line) curves are shown in Figure 3a. We can see the broadband absorption spectrum was composed of three resonant peaks at 1.72, 2.08, and 2.59 THz which are named as peak I, peak II, and peak III for convenience. The three peaks achieved a broadband absorption from 1.68 THz to 2.74 THz with amplitude more than 90%. Figure 3b shows the absorption rate under different polarization of the incident wave. From the figure, we can see that the absorption rate was independent of polarization of the incident wave, which results from the central symmetry of the proposed structure [31].

**Figure 4:**
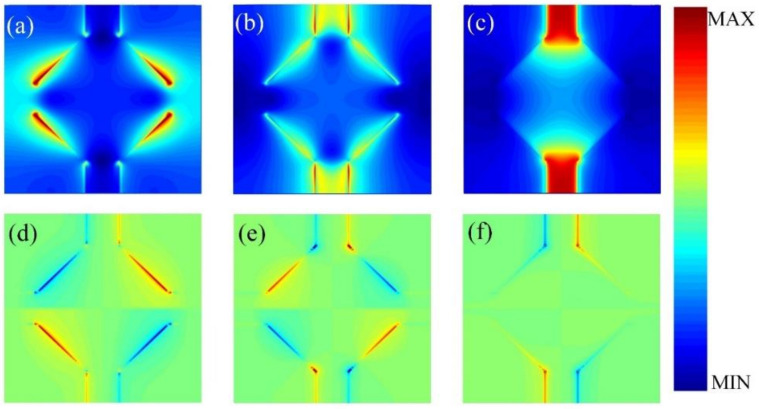
The electric field distribution at (**a**) 1.72 THz, (**b**) 2.08 THz, (**c**) 2.59 THz; the charge distribution at (**d**) 1.72 THz, (**e**) 2.08 THz, (**f**) 2.59 THz.

In order to understand the broadband absorption mechanism clearly, the electric field distribution and charge distribution at 1.72 THz, 2.08 THz, and 2.59 THz were investigated and are illustrated in Figure 4a–f. Figure 4a shows that at 1.72 THz, the electric field enhancement concentrated mainly on the longitudinal graphene strips and the long sides of the octagonal grapheme. Correspondingly, the charge distribution is shown in Figure 4d. It can be observed that opposite charges symmetrical along the y-axis accumulated at the electric field resonance position, which implies that the graphene resonance mode resulted from the resonance between neighbor sides. In Figure 4b, at 2.08 THz, we can see that the electric field resonance was still concentrated on the longitudinal graphene strips and the long sides of the octagonal graphene, but the resonance intensity at the graphene strips was stronger than that in Figure 4a. The electric field enhancement at the long sides of the octagonal graphene became weak and moved close to the longitudinal graphene strips. Moreover, in Figure 4e, the opposite charge distribution at the edge of the octagonal graphene was different from that in Figure 4d. Figure 4c shows that at 2.59 THz, the electric field enhancement was almost entirely concentrated in the longitudinal graphene strips, and by analyzing the charge distribution in Figure 4f, it can be concluded that the strong absorption was caused by the dipole resonance at the longitudinal graphene strips.

The loss A(ω) of terahertz wave at patterned graphene can be obtained by Equation (4) [40]:

(4) $$A(\omega )=\omega \varepsilon^{\prime \prime }{\int_{v}\left|E\right|}^{2}dV$$

where *V*, $\left|E\right|$, and $\varepsilon^{\prime \prime }$ are the volume of graphene, the electric field inside graphene, and the imaginary part of graphene permittivity, respectively. The terahertz wave underwent multiple reflections between the gold film and the patterned graphene to achieve enhanced electromagnetic loss. According to Equation (4), it was the enhanced electric field in the patterned graphene that resulted in the high broadband absorption.

**Figure 5:**
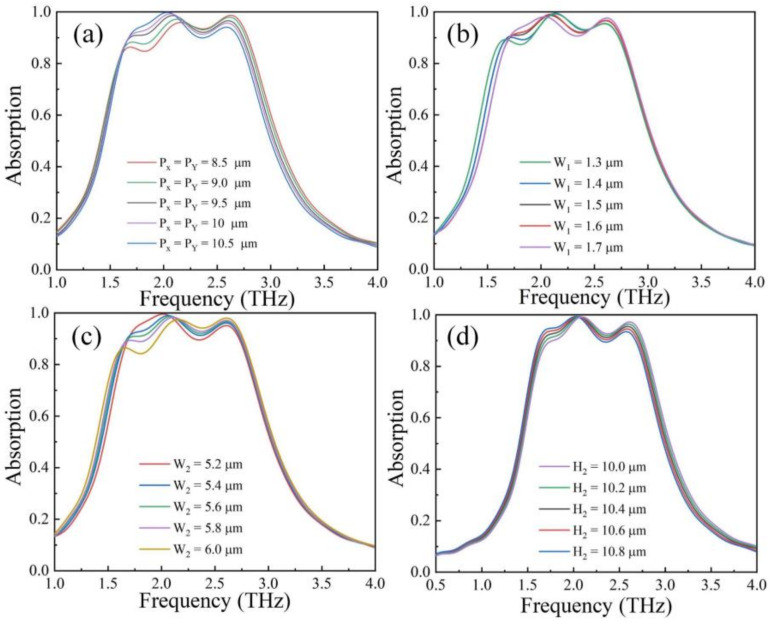
Absorption changes with different (**a**) structural periods, (**b**,**c**) graphene parameters, and (**d**) PDMS thickness.

In fact, changing absorber parameters will also affect the absorption characteristics. In this part, we investigated the influence of different periods, the parameters of the graphene, and the thickness of the PDMS on the absorption properties. When changing the absorber unit period, the absorber absorption effect is shown in Figure 5a. It can be seen that as the period increased from 8.5 μm to 10.5 μm, the intensities of peak I and peak II increased gradually, while the intensity of peak III decreased gradually. As shown in Figure 5b, when the graphene strips width $W_{1}$ changed from 1.3 μm to 1.7 μm, the absorption spectrum changed. It can be observed that absorption at peak I and peak III increased gradually, while peak II absorption rates decreased gradually. Figure 5c shows the change of the absorption spectrum with different graphene length $W_{2}$. It can be observed that with the parameter $W_{2}$ increasing, the absorption at peak I and peak II weakened gradually, while the absorption rate at peak III increased gradually. Figure 5d shows the change of the absorption spectrum as the PDMS thickness: $H_{2}$ increased from 10 μm to 10.8 μm. It can be found that as the thickness of the PDMS increased, the absorption at peak I and peak III showed a weak decrease and increase, respectively, and the peak II absorption was almost unchanged.

**Figure 6:**
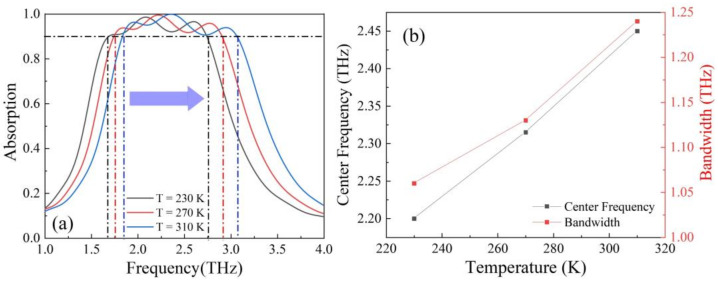
(**a**) The broadband absorption spectra under temperature of 230 K, 270 K, 310 K; (**b**) the changing of center frequencies and bandwidth with temperature.

Furthermore, we studied the effect of STO and graphene on the absorption properties of the absorber above when the Fermi level of graphene was set to 1 eV. Figure 6a shows the absorption of the absorber under different temperatures. We can see that when temperature increased from 230 K to 310 K, three absorption peaks moved to high frequencies. In order to describe the shift of broadband absorption spectra with temperature, we calculated the shift via the center frequency $\left(f_{c}\right)$ expressed as: $f_{c}=\left(f_{L}+f_{H}\right)/2$, where $f_{L}$ is the lowest frequency and $f_{H}$ the highest frequency. Figure 6b shows the changing of center frequencies and bandwidth with temperature. It can be found that by adjusting temperature the center frequency and absorption bandwidth of the broadband, absorption could be tuned from 2.2 THz to 2.45 THz and from 1.06 THz to 1.24 THz, respectively.

**Figure 7:**
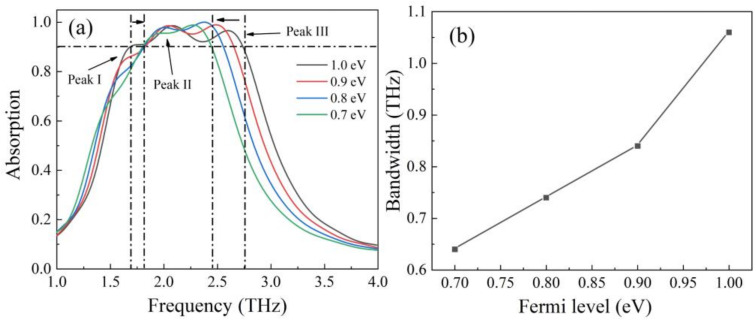
(**a**) The broadband absorption spectrums with the decreased Fermi level from 1 eV to 0.7 eV; (**b**) the relationship between bandwidth and Fermi level.

Figure 7 shows the absorption performance when the Fermi level of grapheme was set to be 1.0–0.7 eV and the STO temperature was maintained at 230 K. From Figure 7a, we can see absorption peaks redshift simultaneously. According to the formula $f\propto {\left(E_{F}\right)}^{\frac{1}{2}}$, the resonance frequency of graphene will decrease with decreased Fermi level [41]. In addition, we can see the absorption intensity of peak I decreased significantly, while peak II and peak III still maintained 95% absorption rate. Therefore, as the Fermi level decreased, the broadband absorption with an absorption rate exceeding 90% was mainly contributed to by peak II and peak III. Since the redshift distance of peak II was smaller than that of peak III, the absorption bandwidth was narrower than before. In order to clearly observe the change of bandwidth with decreasing Fermi level, Figure 7b was given. From the figure, we can see when the Fermi level decreased from 1.0 eV to 0.7 eV, the absorption bandwidth decreased from 1.06 THz to 0.64 THz.

**Figure 8:**
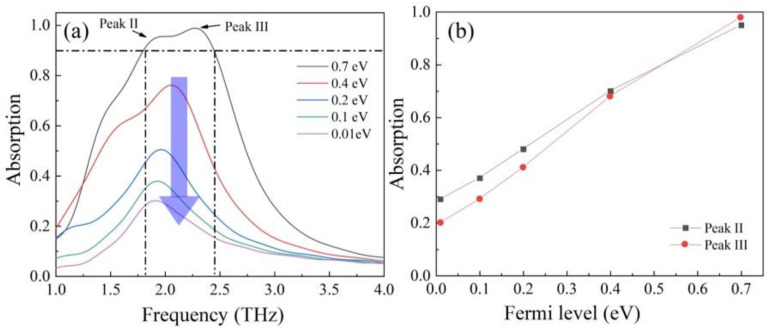
(**a**) The broadband absorption spectrums with the decrease in Fermi level from 0.7 eV to 0.01 eV; (**b**) the MD at peak I and peak II with different Fermi level.

Furthermore, we investigated the influence of Fermi level on absorption intensity. In Figure 8a, we can see when reducing the Fermi level further from 0.7 eV to 0.01 eV, the absorption intensity decreased significantly. Finally, when the graphene was 0.01 eV, there was only one absorption peak with the highest absorption of 0.29 at 1.89 THz. Here we investigated the change of absorption performance at peak I and peak II with the decreased Fermi level and the results are shown in Figure 8b. We can see that with the Fermi level decreasing from 0.7 eV to 0.01 eV, the absorption rate at peak I changed from 0.95 to 0.29 and the absorption rate at peak II changed from 0.98 to 0.20. The modulation depth (MD) can be obtained via formula $\Delta T=[\left(T_{0}-T_{g}\right)/T_{0}]\times 100\%$. Based on the formula, the MD of peak II and peak III was 69% and 79%, respectively. Therefore, the absorber can also achieve the tunable intensity of broadband absorption. This tunable phenomenon can be explained as below. When the Fermi level of graphene is high, graphene is similar to quasi-metal with high conductivity. In this case, electromagnetic waves can excite the localized surface plasmon resonance (LSPR) of graphene, which cause strong efficient absorption [42]. When the Fermi level is reduced to 0.01 eV, the conductivity is too low to support LSPR, so the broadband absorption peak disappears.

In this section, the mechanism of the tunable absorption is studied using the impedance matching theory. The impedance result can be obtained by Equations (5) and (6) [43]:

(5) $$A(\omega )=1-R(\omega )=1-{\left|\frac{Z-Z_{0}}{Z+Z_{0}}\right|}^{2}=1-{\left|\frac{Z_{r}-1}{Z_{r}+1}\right|}^{2}$$

**Figure 9:**
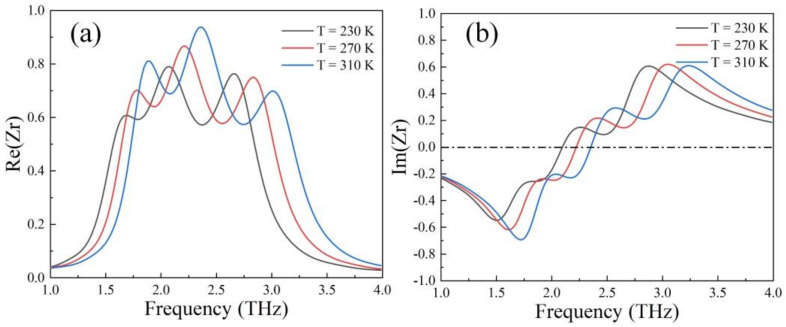
(**a**) Real and (**b**) imaginary part of impedance $Z_{r}$ with increased temperature.

From Equation (5), we can see that the absorption rate A(ω) can be calculated from the absorber impedance $Z_{}$ and the free space impedance $Z_{0}$. In addition, $Z_{r}=Z/Z_{0}$ is the normalized complex impedance. According to Equation (5), $Z_{r}=1$ means that the impedance match between absorber and free space reaches the maximum, thus the perfect absorption effect can be achieved. In addition, $Z_{r}$ can also be obtained by [44]:

(6) Zr=Re(Zr)+Im(Zr)=±(1+S11(ω))2−S212(ω)(1−S11(ω))2−S212(ω)

where $S_{11}(\omega )$ and $S_{21}(\omega )$ are S-parameters. Figure 7 illustrates the influence of device temperature on real and imaginary parts of impedance $Z_{r}$. In Figure 9, at 230 K, in the range of 1.68 THz to 2.74 THz, the real part of impedance is close to 1 and the imaginary part is close to 0, namely, the conditions for realizing impedance matching between relative impedance and free space is achieved and results in high broadband absorption. With the increase of temperature from 230 K to 310 K, both the real part and the imaginary showed simultaneous blueshift. This phenomenon indicates that the region where the absorber impedance was highly matched to the free space impedance moved to high frequency and caused a shift in the broadband absorption frequency.

**Figure 10:**
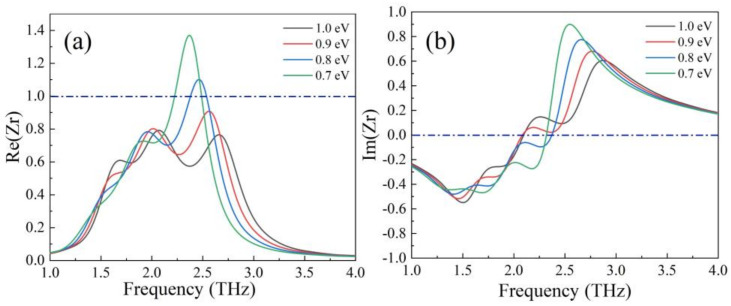
(**a**) Real and (**b**) imaginary parts of impedance $Z_{r}$ with the decrease Fermi level from 1 eV to 0.7 eV.

At the fixed temperature of 230 K, by changing the Fermi level from 1 eV to 0.7 eV, the changes of real and imaginary part of impedance are shown in Figure 10a,b. We can see that as the Fermi level decreased, the frequency range in which the real part of the impedance approaches 1 became narrow. At the same time, the region where the imaginary part approaches 0 became narrow gradually. When the perfect impedance match region became narrow, the absorption bandwidth also reduced. This phenomenon proves that the tuning of the bandwidth of broadband absorption can be achieved by changing the Fermi level of graphene.

**Figure 11:**
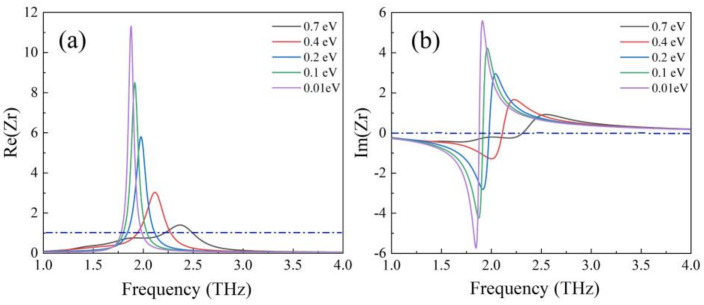
(**a**) Real and (**b**) imaginary parts of impedance $Z_{r}$ with the decrease Fermi level from 0.7 eV to 0.01 eV.

Figure 11 shows the real and imaginary parts of the impedance change when the graphene Fermi level decreased continuously from 0.7 eV to 0.01 eV. We can observe that the real and imaginary part of impedance in the range of 1.81 THz to 2.45 THz gradually deviated from 1 and 0 when the Fermi level decreased. According to Equation (5), this phenomenon leads to a mismatch between the impedance of absorber and that of free space, resulting in a decrease in the absorption rate. Therefore, with the decrease of Fermi level, the absorption rate of the device decreases gradually.

**Figure 12:**
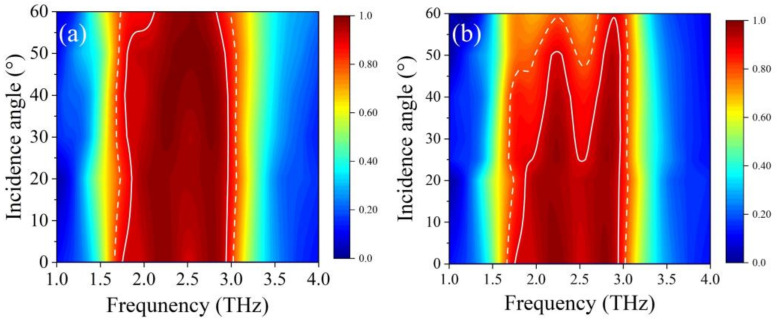
The absorption spectrum changed as the incident angle of (**a**) TE and (**b**) TM waves increased from 0° to 55°.

In this part, we discuss the influence of incident angles of the terahertz wave on absorber performance. The Fermi level and temperature were set to be 1.2 eV and 260 K, respectively. As shown in Figure 12a,b, in the area within the white solid line and dotted line, the absorption rate reached 90% and 80%, respectively. For the incidence of TE polarized wave, within the angle of 55°, we can see that the width of the white solid line area was almost unchanged, which means the absorption peak maintained a broadband absorption of more than 90%. For TM polarized wave incidence, the absorber maintained high broadband absorption rate within 20°. According to the dotted line, even when the incidence angle increased to 45°, the amplitude of broadband absorption was still beyond 80%. The results above indicate that the absorber is not sensitive to incidence angles.

Finally, the comparison of our design with the previous similar absorbers is made and shown in Table 1. Compared with the absorber in Ref. [31], our work can realize much wider absorption bandwidth and higher MD. Compared with the dual-function absorbers in Refs. [28,33,45], our design can realize the three tunable functions, and the absorption bandwidth of our design is larger than that of the absorber in Ref. [33]. The MD of our design is higher than that of the absorber in Ref. [28].

**Table 1:** The comparison between references and our design.

References	Absorber Bandwidth Regulation	Modulation Depth	Center Frequency Regulation	Tunable Materials
[28]	Unrealized	65%	0.3–0.43	STO and Graphene
[31]	0.225–0.34 THz	30%	2.665–3.69 THz (Adjusting STO) and 3.265–4.82 THz (Adjusting BDS)	STO and BDS
[33]	0.12–0.38 THz	Unrealized	Broadband: 0.8 to 0.97 THz; Narrowband: 0.56 to 0.84 THz	STO and Graphene
[45]	Broadband (1.3 THz)—narrowband	~54.5% (broadband) and 90% (narrowband)	Unrealized	Graphene and VO_2_
**This work**	0.64–1.24 THz	69–79%	2.2–2.45 THz	STO and Graphene

## 4. Conclusions

In summary, a broadband multifunctional absorber composed of graphene and STO was proposed and investigated theoretically based on the FDTD algorithm. The results show that, when tuning the temperature, center frequency modulation of broadband absorption can be realized. When the Fermi level was changed from 1 eV to 0.7 eV, the absorber realized the modulation of absorption bandwidth. As the Fermi level continued to change from 0.7 eV to 0.01 eV, the intensity of absorption rate could be tuned. The impedance matching theory was used to analyze the mechanism of tunable broadband absorption. In addition, with the different polarization angle of terahertz wave, the absorption rate did not change and the absorber achieved a good absorption performance within the large oblique incidence angle. The absorber proposed in this work can provide potential applications for future multifunctional absorbers and optical switches.

## Data Availability

The data are included in the main text.
